# Potential Metabolic Biomarkers to Identify Interstitial Lung Abnormalities

**DOI:** 10.3390/ijms17071148

**Published:** 2016-07-16

**Authors:** Yong Tan, Dongmei Jia, Zhang Lin, Baosheng Guo, Bing He, Cheng Lu, Cheng Xiao, Zhongdi Liu, Ning Zhao, Zhaoxiang Bian, Ge Zhang, Weidong Zhang, Xinru Liu, Aiping Lu

**Affiliations:** 1 Institute of Basic Research in Clinical Medicine, China Academy of Chinese Medical Sciences, Beijing 100700, China; tcmtanyong@126.com (Y.T.); cheng0816@163.com (C.L.); zhaon2003@163.com (N.Z.); 2 Traditional Chinese Medicine Division, Science Press, Beijing 100717, China; jdm3213@163.com; 3 School of Pharmacy, Second Military Medical University, Shanghai 200433, China; linzhang19890211@163.com (Z.L.); wdzhangy@hotmail.com (W.Z.); 4 Institute for Advancing Translational Medicine in Bone & Joint Diseases, School of Chinese Medicine, Hong Kong Baptist University, Hong Kong 999077, China; boris.g.guo@gmail.com (B.G.); hebinghb@gmail.com (B.H.); liuzhongdi1980@sina.com (Z.L.); bzxiang@hkbu.edu.hk (Z.B.); zhangge@hkbu.edu.hk (G.Z.); 5 Institute of Clinical Medicine, China-Japan Friendship Hospital, Beijing 100029, China; xc2002812@126.com; 6 E-Institutes of Shanghai Municipal Education Commission, Shanghai 201203, China

**Keywords:** interstitial lung abnormalities, biomarkers, serum metabolic profiles

## Abstract

Determining sensitive biomarkers in the peripheral blood to identify interstitial lung abnormalities (ILAs) is essential for the simple early diagnosis of ILAs. This study aimed to determine serum metabolic biomarkers of ILAs and the corresponding pathogenesis. Three groups of subjects undergoing health screening, including healthy subjects, subjects with ILAs, and subjects who were healthy initially and with ILAs one year later (Healthy→ILAs), were recruited for this study. The metabolic profiles of all of the subjects’ serum were analyzed by liquid chromatography quadruple time-of-flight mass spectrometry. The metabolic characteristics of the ILAs subjects were discovered, and the corresponding biomarkers were predicted. The metabolomic data from the Healthy→ILAs subjects were collected for further verification. The results indicated that five serum metabolite alterations (up-regulated phosphatidylcholine, phosphatidic acid, betaine aldehyde and phosphatidylethanolamine, as well as down-regulated 1-acylglycerophosphocholine) were sensitive and reliable biomarkers for identifying ILAs. Perturbation of the corresponding biological pathways (RhoA signaling, mTOR/P70S6K signaling and phospholipase C signaling) might be at least partially responsible for the pathogenesis of ILAs. This study may provide a good template for determining the early diagnostic markers of subclinical disease status and for obtaining a better understanding of their pathogenesis.

## 1. Introduction

Interstitial lung abnormalities (ILAs), the asymptomatic or subclinical lung disease stage often present in the cigarette smoking, aging, and male population, are characterized by mild radiologic changes in the pulmonary interstitium [1,2,3,4,5,6]. The widespread use of high-resolution computed tomography (HRCT) in clinical and research settings has increased the detection of ILAs [7]. Accumulating evidence has suggested that individuals with ILAs have reductions in total lung capacity, functional limitations, histopathologic changes, and molecular profiles similar to those observed in patients with clinically significant interstitial lung disease (ILD) [1,6,8,9]. Growing findings have demonstrated that ILAs are risk factors for ILD, emphysema, pulmonary fibrosis and lung cancer. Specifically, ILAs represent the early stages of subclinical ILD or idiopathic pulmonary fibrosis (IPF) [3,10,11,12]. A clinical survey showed that approximately 37% of ILAs progressed to IPF in cigarette smokers [13]. ILAs are the main reason for the reduced lung capacity in paraseptal emphysema [5,6,9]. More seriously, ILAs are related to an increased risk of lung cancer [14]. A study showed that higher ILAs scores were associated with shorter overall survival, indicating that ILAs could be a marker of shorter survival in advanced non-small-cell lung cancer (NSCLC) [15]. As of today, however, ILAs lack simple and reliable diagnostic indicators, and their pathogenesis is unclear. These findings provide an important motivation to seek sensitive biomarkers in the peripheral blood for identifying ILAs and to reveal the pathogenesis of ILAs.

Increasing evidence has shown that lung function impairment in adults was associated with systemic metabolic disorders [16,17,18,19,20]. Therefore, exploring the pathogenesis of this impairment from the systemic metabolism becomes an important study focus. Metabolomics technology provided technical support for this study. Metabolomics is the quantitative measurement of the dynamic multi-parametric metabolic response of living systems to pathophysiological stimuli or genetic modifications [21]. By measuring and mathematically modeling changes in the products of metabolism found in biological fluids and tissues, metabolomics offers fresh insight into disease [22]. As a monitoring tool of human metabolism and endogenous biochemical pathways [23], metabolomics has been successfully applied to identify potential biomarkers and to reveal the metabolic changes and the underlying mechanisms of a great variety of diseases [24,25,26,27]. It has also been used in some respiratory illnesses, such as chronic obstructive pulmonary disease (COPD) and lung cancer [28,29].

In this study, a metabolomics-based liquid chromatography quadruple time-of-flight mass spectrometry (LC–Q–TOF–MS) technique was used for demonstrating the serum metabolic characteristics. We predicted ILAs biomarkers on the basis of comparing the metabolic data of subjects with ILAs and healthy control subjects and, subsequently, we verified the predicted results using metabolomic data from the subjects who were disease-free initially and then one year later suffered from ILAs (Healthy→ILAs). We aimed to determine the sensitive and reliable biomarkers responsible for ILAs and to reveal the corresponding metabolic pathways.

## 2. Results

### 2.1. Baseline Characteristics of Study Subjects

The characteristics of the enrolled subjects, including sex, age, smoking status, and results of blood routine and biochemical tests, are shown in Table 1. The smoking rates of the ILAs group, initial stage (healthy) group and outcome stage (ILAs) group were much higher than that of the control group. There was no significant difference in other examination indicators among the groups.

### 2.2. Evaluation of the Repeatability and Stability of the LC–Q–TOF–MS Method

The repeatability was evaluated through continuously injecting extracts from six aliquots of a random blood sample [30,31]. Five common extracted ion chromatograms (EICs) shared by these injections were selected on the basis of their different chemical polarities and *m*/*z* values. The relative standard derivations (RSDs) of these peaks were 4.13%–13.13% for peak areas and 0.04%–0.98% for retention times.

The stability for the large-scale sample analysis was demonstrated by the test of pooled quality control (QC) samples. The principal component analysis (PCA) result indicated that the QC samples were tightly clustered. In addition, the peak areas, retention times and mass accuracies of five selected EICs from five QC samples also showed good system stability. The RSDs of the five peaks were 4.94%–14.88% for peak areas, 0.03%–1.10% for retention times and 0.14 × 10^−4^%–0.76 × 10^−4^% for mass accuracies. The result showed that the large-scale sample analysis had no apparent effect on the reliability of the data.

### 2.3. Identification of the Differential Metabolites in Interstitial Lung Abnormalities (ILAs)

**Figure 1:**
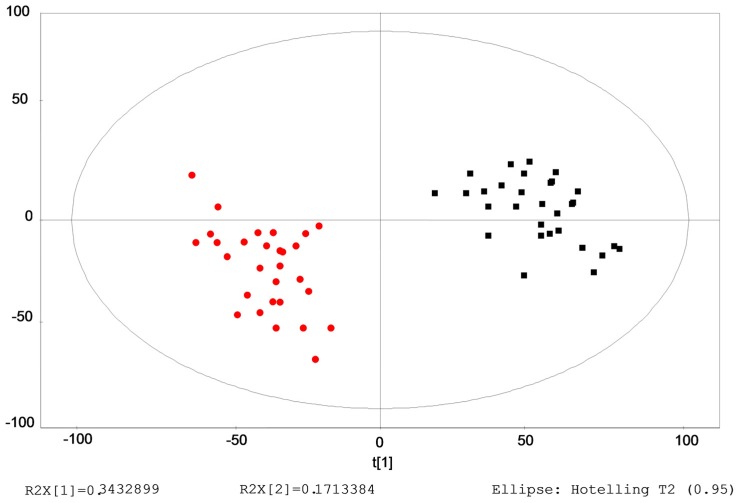
Multiple pattern recognition of metabolites in the control and ILAs groups. PLS-DA score plot (*n* = 59): Control group (■); and ILAs group (●).

Typical base peak chromatograms (BPCs) of serum samples were obtained from the control and ILAs groups. Multiple pattern recognition methods of partial least squares discriminant analysis (PLS-DA) were adopted on the basis of the metabolic changes in these subjects as revealed by BPCs. These methods facilitated the classification of the metabolic phenotypes and enabled us to identify the differential metabolites. As shown in score plots (Figure 1), there was obvious separation between the control and ILAs groups.

Eleven metabolites were identified in the ILAs subjects when compared to those in healthy control subjects (Table 2). Representative value (RV), a value used for showing the power of the metabolite to reflect the abnormal state in the disease, was reduced in sequence from phosphatidylcholine (PC)(28:1) to betaine aldehyde (BA), which implied that PC(28:1) was the most representative metabolite for indicating the characteristic of ILAs.

### 2.4. Verification of the Identified Metabolites by Healthy→ILAs Subjects

**Figure 2:**
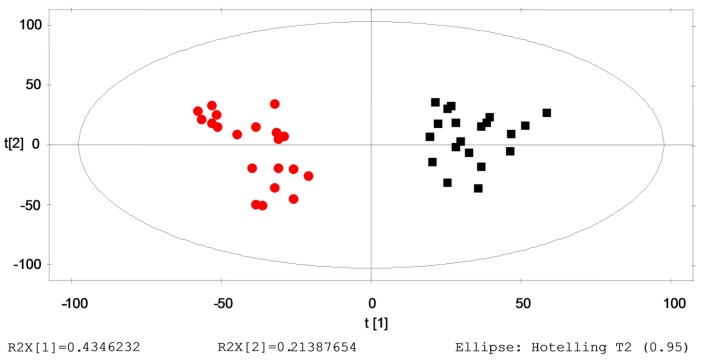
Multiple pattern recognition of metabolites in initial stage (healthy) and outcome stage (ILAs) groups. PLS-DA score plot (*n* = 40): Initial stage (healthy) group (■); and outcome stage (ILAs) group (●).

Based on the metabolic changes in the subjects who were healthy initially and who subsequently were identified with ILAs one year later, we verified the predicted metabolites responsible for the progression from healthy to ILAs. Multiple pattern recognition methods were adopted. PLS-DA score plots showed obvious separation between the initial stage (healthy) group and outcome stage (ILAs) group, as shown in Figure 2.

Compared with the initial stage (healthy) subjects, 9 metabolites were identified in the outcome stage (ILAs) subjects (Table 3).

**Figure 3:**
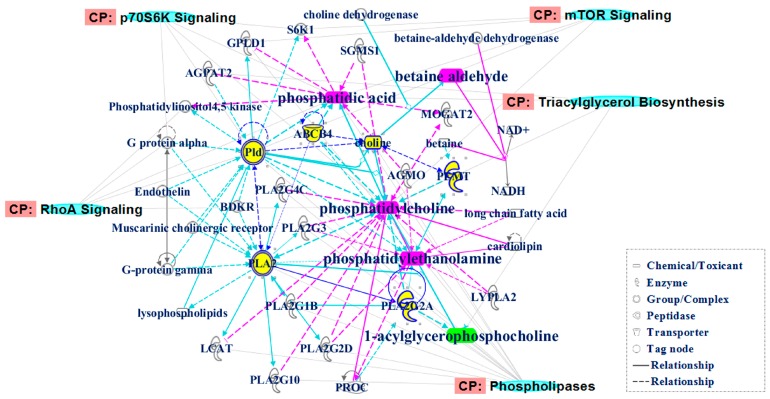
Biological network and canonical pathways related to the identified common metabolites. In the network, molecules are represented as nodes, and the biological relationship between two nodes is represented as a line. Red symbols represent up-regulated metabolites; green symbols represent down-regulated metabolites; yellow symbols are the high link molecules from the Ingenuity Knowledge Database, while the blue symbols represent canonical pathways that are related to the identified specific metabolites. Solid lines between molecules show a direct physical relationship between molecules, while dotted lines show indirect functional relationships.

Through the comparison between these metabolites and the predicted metabolites, we found that up-regulated phosphatidylcholine (PC), phosphatidic acid (PA), phosphatidylethanolamine (PE) and betaine aldehyde (BA), as well as down-regulated 1-acylglycerophosphocholine (1-acyl-GPC), were their common metabolites. The correlations between the metabolites were identified using IPA. As shown in Figure 3, these metabolites are correlated with some canonical pathways, such as phospholipases, triacylglycerol biosynthesis, RhoA signaling, P70S6K signaling, mTOR signaling, etc. (Table S1). The associated biological functions were focused on lipid metabolism, carbohydrate metabolism and energy production.

## 3. Discussion

ILAs are regarded as risk factors for ILD, emphysema, pulmonary fibrosis and even lung cancer. Early identification and removal of this risk factor are crucial for preventing disease progression. Currently, the identification of ILAs depends mainly on HRCT, which is not widely available for health screening in rural areas, and the pathogenesis of ILAs is not clear. Therefore, this study aimed to explore sensitive and reliable biomarkers to identify ILAs and the pathogenesis of ILAs. Based on ILAs subjects from large-sample health-screening populations, using LC–Q–TOF–MS and molecular network analysis, we discovered for the first time the serum biomarkers and corresponding pathogenesis of ILAs (Figure 3). Five metabolites, including up-regulated phosphatidylcholine (PC), phosphatidic acid (PA), phosphatidylethanolamine (PE) and betaine aldehyde (BA), as well as down-regulated 1-acylglycerophosphocholine (1-acyl-GPC), were identified as biomarkers for ILAs. Accordingly, disturbances in RhoA signaling, mTOR/P70S6K signaling and phospholipase metabolism could be partially responsible for ILAs pathogenesis.

PC is a major component of biological membranes in higher eukaryotes, and it can be secreted by specialized tissues for important extracellular tasks. PC levels can be regulated by multifarious phospholipases, such as phospholipase A (PLA) and phospholipase D (PLD), which catalyze the hydrolysis of PC [32]. In the lungs, PC is synthesized within the alveolar type II epithelium, and it is secreted into the alveoli as the major phospholipid component of pulmonary surfactant [33,34]. PC molecular species in lung surfactant are composed relative to respiratory rate and lung development [35]. The evidence indicated that the hepatic and plasma homeostasis of choline and PC was correlated with lung function and inflammation [36]. Increased PLA2 and lyso-PC levels were associated with surfactant dysfunction in lung contusion injury in mice [37]. The up-regulated PC in this study might be partially associated with the epithelial injury in ILAs. PA is the most critical metabolite generated in PC hydrolysis that is catalyzed by PLD, and it can be metabolized into lyso-PA and diacylglycerol, which function as first- and second-generation messengers, respectively [38]. PA is involved in a variety of cellular functions, such as cell proliferation, cytoskeleton organization, morphogenesis and vesicle trafficking [38,39,40]. In the lungs, PA and its metabolic products play central roles in modulating endothelial and epithelial cell functions [41]. PA was capable of inducing lung endothelial cell cytotoxicity, suggesting a possible bioactive lipid-signaling mechanism of the microvascular disorders encountered in IPF [42]. PA signaling mediated lung cytokine expression and lung inflammatory injury after hemorrhage in mice [43]. Wound-induced epithelial cell motility might be mediated by PA signaling [44]. Up-regulated PA in this study was also partially responsible for the epithelial injury in ILAs. Biological pathway analysis revealed that PC and PA were mainly involved in some bio-pathways, such as RhoA signaling, mTOR signaling and P70S6K signaling. A previous study showed that RhoA signaling modulated cyclin D1 expression in human lung fibroblasts and was implicated in IPF [45]. The mTOR complex is a highly conserved intracellular serine/threonine kinase. The mTOR expression in pulmonary fibrosis patients was significantly correlated with the fibrosis score and decreased lung function, indicating that it might be related to the prognosis of pulmonary fibrosis [46]. Furthermore, inhibiting mTOR activation could enhance autophagy and suppress fibrotic markers in IPF [47]. The mTOR signaling pathway is a key regulator of cell growth and proliferation [48]. PA directly interacted with the domain in mTOR, and this interaction was positively correlated with mTOR’s ability to activate downstream effectors [49]. P70S6K is the major downstream molecule of mTOR, and it could be activated by mTOR [50,51,52]. PA could activate P70S6 independent of mTOR [53].

Betaine aldehyde is the degradation product of choline via the choline degradation pathway. An increased betaine aldehyde level indicates that the hydrolysis reaction of PC by PLD might be activated excessively. Furthermore, the increase could convert into betaine through dehydrogenation. Disturbance of this series of reactions can aggravate the metabolic disorders of PC. PE is also a class of phospholipids found in biological membranes. Synthesis of PC and PE occurred in relation to the concentration of membrane-bound diacylglycerols of rat lung microsomes [54]. PE-binding protein 4 promoted lung cell proliferation and invasion via the PI3K/Akt/mTOR axis [55]. Up-regulated betaine aldehyde and PE were also partially responsible for epithelial injury in ILAs. 1-acyl-GPC is one of the metabolites of PC in the catalysis of PLA2, and it is involved in phospholipase metabolism and triacylglycerol biosynthesis, together with PC and PA. Secretory PLA2 (sPLA2) is an emerging class of mediators of inflammation. These enzymes accumulate in the plasma and other biological fluids of patients with inflammatory, autoimmune and allergic diseases. Lung mast cells are a source of sPLA2 [56]. sPLA2s are secreted at low levels in normal airways, and they tend to increase during inflammatory lung diseases (e.g., bronchial asthma, chronic obstructive pulmonary disease, interstitial lung fibrosis, and sarcoidosis) as the result of plasma extravasation and/or local production. Thus, sPLA2s could play a major role in inflammatory lung diseases by acting as a proinflammatory connection between macrophages and mast cells [57]. In this study, 1-acyl-GPC was down-regulated, which might have been result of the suppressed hydrolysis of PC by PLA2.

Taken together, five metabolite alterations, including up-regulated PC, PA, betaine aldehyde and PE, as well as down-regulated 1-acyl-GPC, served as reliable biomarkers to identify ILAs. Accordingly, altered RhoA signaling, mTOR/P70S6K signaling and phospholipase metabolism could be partially responsible for ILAs pathogenesis. This study provided a good template for determining the early diagnostic markers of subclinical disease status and a better understanding of their pathogenesis. A limitation of this study was that most of the recruited subjects were office workers; their prevalence of ILAs might be lower than that in manual workers. It is necessary to expand the sample size in future studies.

## 4. Materials and Methods

### 4.1. Study Population

In total, 8401 individuals (5928 men and 2473 women) underwent health screenings at Hangxin Hospital, Beijing, China, from 2011 to 2013. Specifically, 2763 individuals underwent health screenings in 2011, 2706 in 2012 and 2932 in 2013.

ILAs were identified by HRCT. Some subjects were excluded for the following reasons: age ≥75 years old or age ≤18 years old; other causes of chronic lung diseases or mixed etiologies (asthma, emphysema, cor pulmonale, chronic obstructive pulmonary disease, lung fibrosis, pneumoconiosis and lung cancer); complications such as cardiovascular and cerebrovascular diseases, chronic liver disease, gastrointestinal disease, nephropathy, and metabolic syndrome; domestic and occupational environmental exposure histories; and intake of certain drugs (bleomycin, cyclosporine, sirolimus, mycophenolic acid, amiodarone, Dilantin, statins and azathioprine).

**Figure 4:**
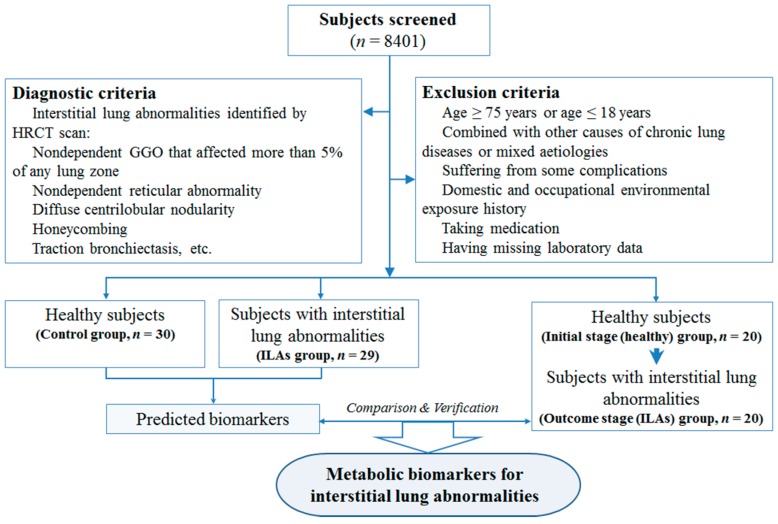
Clinical trial design flow diagram.

Consequently, 79 subjects comprised the cohort for this study, including 30 healthy subjects (control group: 22 men and 8 women), 29 ILAs subjects (ILAs group: 22 men and 7 women), and 20 Healthy→ILAs subjects (initial stage (healthy) group and outcome stage (ILAs) group: 16 men and 4 women) (Figure 4). This study was approved by the Ethics Committee at the Institute of Basic Research in Clinical Medicine, China Academy of Chinese Medical Sciences, and was conducted according to the standards of the Declaration of Helsinki. Written informed consent was obtained from the participants.

### 4.2. Questionnaire

All of the subjects were asked to complete a questionnaire in regard to their symptoms, medical histories, drug usage, smoking histories, and domestic and occupational environmental exposure histories. The questions on smoking included the frequency of tobacco consumption per week and the usual amount that was consumed.

### 4.3. Peripheral Blood Sampling and Biochemical Testing

Fasting blood samples were drawn via venipuncture from the study subjects by experienced clinical nurses. After standing for 2 h at 4 °C, the blood samples were centrifuged at 3500× *g* for 15 min. The obtained serum was divided into two parts: one part was used for blood routine examination and biochemical examination according to the manufacturers’ instructions of the respective commercial test kits, including white blood cell count (WBC), red blood cell count (RBC), blood platelet count (PLT), lymphocyte count (LY), neutrophil count (NE), hemoglobin count (HGB), alanine aminotransferase(ALT), aspartate aminotransferase (AST), creatinine (CRE) and serum uric acid (SUA). The remaining 100 μL of serum was mixed with 200 μL of acetonitrile, and the mixture was vortexed for 30 s. After centrifugation at 9560× *g* for 10 min at 4 °C, the supernatant was stored at −80 °C for LC/MS analysis.

A random blood sample (6 mL) was divided into six parts and extracted by the same method. These six samples were continuously injected to validate repeatability of the sample preparation method. 20 μL from each blood sample was pooled to generate a pooled QC sample and aliquots of 100 μL of this pooled sample were extracted by the same method. This pooled sample was used to provide a representative “mean” sample containing all analytes that was encountered during the analysis, and it was used to validate stability of LC–MS system.

### 4.4. High-Resolution Computed Tomography (HRCT) Examinations

HRCT plays a crucial role in the diagnosis, prognosis, quantification and monitoring of ILAs. It provides a definite noninvasive diagnosis in typical findings and helps to obtain the most accurate diagnosis in a multidisciplinary discussion in equivocal cases [58]. In this study, all of the subjects underwent HRCT examinations. The HRCT findings were categorized on a 3-point scale (0 = no evidence of ILAs, 1 = equivocal for ILAs, 2 = ILAs) by a sequential reading method previously reported. ILAs scores of 2 indicated the presence of ILAs. Findings equivocal for ILAs were defined as focal or unilateral ground-glass opacity (GGO), focal or unilateral reticulation, and patchy GGO (<5% of the lung). An ILAs was defined as nondependent GGO that affected more than 5% of any lung zone, nondependent reticular abnormality, diffuse centrilobular nodularity with GGO, honeycombing, traction bronchiectasis, nonemphysematous cysts, or architectural distortion. Centrilobular nodularity alone was not considered to be evidence of ILAs.

### 4.5. LC–Q–TOF–MS Analysis

LC–Q–TOF–MS analysis was undertaken using an Agilent-1200 LC system coupled with an electrospray ionization (ESI) source (Agilent Technologies, Palo Alto, CA, USA) and an Agilent-6520 Q-TOF mass spectrometer. Separation of all of the samples was performed on an Eclipse plus C18 column (1.8 µm, 3.6 mm × 100 mm, Agilent) with a column temperature set at 45 °C. The flow rate was 0.3 mL/min, and the mobile phase consisted of ultrapure water with 0.1% formic acid and acetonitrile. The gradient program was as follows: 2% acetonitrile for 0–1.5 min; 2%–100% acetonitrile for 1.5–13 min; a wash with 100% acetonitrile for 13–16 min; and a re-equilibration step for 5 min. The sample injection volume was 2 µL.

Mass detection was performed in the positive ion mode with the following settings: drying gas (N2) flow rate, 8 L/min; gas temperature, 330 °C; pressure of nebulizer gas, 35 psig; Vcap, 4000 V; fragmentor, 160 V; skimmer, 65 V; and scan range, *m*/*z* 50–1200. All of the analyses were acquired using the instrument mass spray to ensure accuracy and reproducibility. Leucine encephalin was used as the instrument reference mass (*m*/*z* 556.2771) at a concentration of 50 fmol/μL with a flow rate 40 μL/min. The MS/MS analysis was acquired in targeted MS/MS mode with collision energy from 10 to 40 V.

### 4.6. Sequence Analysis

The stability of the sequence analysis was monitored by the pooled QC sample analysis at the beginning, at the end and randomly throughout the analytical run. The typical batch sequence of the serum samples consisted of the consecutive analysis of 1 QC serum sample (at the beginning of the study), followed by 6 unknown serum samples, and then 1 QC serum sample, before running another 6 unknown serum samples, etc. In the meantime, the samples were analyzed in a random order per normal, good practices. An identical sequence was repeated to complete the total set of injections (*n* = 93, including QCs) analyzed in less than 1 day, as described in previous studies [31].

### 4.7. Data Processing and Statistical Analysis

The LC–MS raw data were exported by the Agilent Mass Hunter Qualitative Analysis Software (Agilent Technologies). The parameters were optimized to improve the extraction of ion information, and the following parameters were chosen: the *m*/*z* values ranged from 80 to 1000, peak filters were set to a centroid height exceeding 100 counts, and compound filters set the base peak to more than 1000 counts. This processing step created MHD files that contained compound IDs (based on neutral mass and retention times), and further processing was conducted with the Mass Profiler software (version B.02.00, Agilent Technologies), which aligns mass features across multiple LC–MS data files. The mass-clustering window was 5 ppm, and the retention time-clustering window was 0.1 min. In this study, the number of signals was 6169 in the positive mode. The sum of the ion peak areas within each sample was normalized to 10,000. PLS-DA was used for the metabolic profile. Multivariate analysis was performed using SIMCA-P software (Umetrics AB, Umeå, Sweden), version 11. The SAS statistical package (order no. 195557), version 9.1.3, was used for the statistical analysis. The attribute data were analyzed using χ-square test. The measurement data obtained indicated a normal distribution. Comparisons between multiple groups were analyzed using analysis of variance. *p* < 0.05 was regarded statistically significant.

### 4.8. Molecular Network Analysis

Molecular networks for the candidate metabolites were built and analysis of bio-functions and canonical pathways were conducted by using the Ingenuity Pathway Analysis system (IPA, Ingenuity^®^ Systems, http://www.ingenuity.com), to gain insight into the typical metabolic alterations associated with the biomarkers and the mechanisms relevant to ILAs.

### 4.9. Prediction of Metabolites Indication Ability

Human protein–protein interaction (PPI) data and enzyme-metabolite interaction (EMI) data were retrieved respectively from the HPRD (http://www.hprd.org/), BioGRID databases (http://thebiogrid.org/) and HMDB database (http://www.hmdb.ca/). PPIs and EMIs supported by at least one wet experiment study were regarded confident and were selected for further analysis. Finally, 304705 PPIs and 452985 EMIs were used in this analysis. It has been hypothesized that changes in metabolites represent changes in the enzymes that involve in catalyzing the metabolites. Due to the changes in enzymes as a result of deregulating upstream pathways in diseases, the metabolites can be used to show the internal molecular abnormal state of the disease. Representative value (RV) is defined as the power of the metabolite to reflect the abnormality of the disease. RV uses the fold change in the metabolite, the number of enzymes catalyzing the metabolite and the importance of every enzyme to evaluate the indicative ability of the metabolite for the disease. RV is calculated by the following arithmetic formula:

(1) $$RV_{m}=\frac{FC_{m}\sum_{i=1}^{n_{e}}EP_{i}}{\sum_{j=1}^{n_{m}}(FC_{m_{j}}\sum_{i=1}^{n_{e}}EP_{i})}$$

where $RV_{m}$ refers to the representative value of the metabolite m; $EP_{i}$ refers to the network power of the enzyme i that involves in catalyzing the metabolite *m*. (the network power is evaluated by the protein–protein interaction (PPI) network degree); $n_{e}$ refers to the number of enzymes involving in catalyzing the metabolite m; $FC_{m}$ refers to the fold change value of metabolite m in the disease compared with the normal state; and $n_{m}$ refers to the number of deregulated metabolites in the disease.

## Figures and Tables

**Table 1:** Characteristics among the groups of the enrolled subjects.

Indicators/Groups	ILAs	Initial Stage (Healthy)	Outcome Stage (ILAs)	Control
Sex (M/F)	22/7	16/4	16/4	22/8
Age (years)	63.90 ± 6.04	60.70 ± 10.00	61.20 ± 9.47	61.11 ± 7.53
Smoking rate (%)	48.28 *	50.00 *	50.00 *	18.95
WBC (10^9^/L)	6.31 ± 1.25	6.51 ± 1.21	6.45 ± 1.38	5.92 ± 1.21
LY (10^9^/L)	1.93 ± 0.54	1.91 ± 0.50	1.93 ± 0.62	1.82 ± 0.46
NE (10^9^/L)	4.29 ± 0.97	4.29 ± 0.96	4.41 ± 1.19	3.79 ± 1.07
RBC (10^12^/L)	4.72 ± 0.33	4.79 ± 0.47	4.80 ± 0.39	4.68 ± 0.45
HGB (g/L)	141.97 ± 11.61	145.05 ± 12.69	141.95 ± 13.29	141.38 ± 10.96
PLT (10^9^/L)	190.14 ± 41.78	200.50 ± 52.00	203.35 ± 51.09	205.82 ± 46.46
ALT (mmol/L)	18.79 ± 7.61	21.85 ± 6.68	16.90 ± 5.15	18.15 ± 8.48
AST (mmol/L)	23.31 ± 6.48	22.15 ± 3.56	22.05 ± 3.30	25.00 ± 14.16
CRE (mmol/L)	76.52 ± 19.56	79.2 ± 11.22	68.45 ± 11.39	70.38 ± 13.02
SUA (μmol/L)	329.79 ± 70.61	341.15 ± 85.76	318.30 ± 85.42	304.54 ± 57.58

The comparisons of clinical indicators among the ILAs group, initial stage (healthy) group, outcome stage (ILAs) group and control group. Chi-square test was used for count variables analysis. Unpaired t-test was applied to continuous variables analysis, and the data are expressed as the mean ± SD when appropriate (95% CI). The ILAs group, initial stage (healthy) group and outcome stage (ILAs) group vs. control group respectively: * *p* < 0.01.

**Table 2:** Identified differential metabolites in the interstitial lung abnormalities (ILAs) group.

*n*	Rt (min)	Exact Mass	Formula	Compound	Fold Changes	RV
1	7.3660	675.4839	C_36_H_70_NO_8_P	Phosphatidylcholine (PC)(28:1)	7.4118	0.0511
2	11.6928	674.4886	C_37_H_71_O_8_P	Phosphatidic acid (PA)(34:1)	12.0640	0.0437
3	11.798	467.3012	C_22_H_46_NO_7_P	1-Acylglycerophosphocholine (1-acyl-GPC)	−17.2178	0.0283
4	8.8195	776.7257	C_50_H_96_O_5_	Triacylglycerol	8.9617	0.0187
5	11.8360	715.5152	C_39_H_74_NO_8_P	Phosphatidylethanolamine (PE)(34:2)	3.5377	0.0166
6	12.3754	148.0194	C_5_H_8_O_3_S	2-Keto-4-methylthiobutyric acid	−19.2816	0.0033
7	9.3124	116.0837	C_6_H_12_O_2_	Caproic acid	2.83241	0.0021
8	8.5394	100.0888	C_6_H_12_O	Caproaldehyde	3.6839	0.0013
9	8.7894	128.1201	C_8_H_16_O	Octanal	4.2036	0.0012
10	7.5562	102.0919	C_5_H_12_NO	Betaine aldehyde (BA)	2.9631	0.0005
11	7.6113	72.0575	C_4_H_8_O	2-Butanone	8.4656	0

Fold change value refers to “ILAs group vs. control group” change values.

**Table 3:** Identified differential metabolites in the outcome stage (ILAs) group vs. initial stage (healthy) group.

*n*	Rt (min)	Exact Mass	Formula	Compound	Fold Changes
1	14.4294	690.5223	C_45_H_70_O_5_	Diacylglycerol	−9.9068
2	11.6928	674.4886	C_37_H_71_O_8_P	PA(34:1)	1.4748
3	11.8360	715.5152	C_39_H_74_NO_8_P	PE(34:2)	7.3538
4	7.3660	675.4839	C_36_H_70_NO_8_P	PC(28:1)	1.6725
5	11.798	467.3012	C_22_H_46_NO_7_P	1-Acylglycerophosphocholine (1-acyl-GPC)	−12.9007
6	1.9809	274.2297	C_19_H_30_O	3-Oxosteroid	11.3056
7	6.7841	121.0891	C_8_H_11_N	Phenylethylamine	−3.3337
8	7.5562	102.0919	C_5_H_12_NO	Betaine aldehyde	1.1937
9	8.7372	202.1205	C_10_H_18_O_4_	Sebacic acid	10.5327

Fold change value refers to the “outcome stage (ILAs) group vs. initial stage (healthy) group” change value.

## Supplementary Materials

Supplementary materials can be found at http://www.mdpi.com/1422-0067/17/7/1148/s1.

## Abbreviations

The following abbreviations are used in this manuscript:

1-Acyl-GPC	1-Acylglycerophosphocholine
ALT	Alanine aminotransferase
AST	Aspartate aminotransferase
AUC	Area under the curve
BA	Betaine aldehyde
BPCs	Base peak chromatograms
COPD	Chronic obstructive pulmonary disease
CRE	Creatinine
EICs	Extracted ion chromatograms
EMI	Enzyme-metabolite interaction
ESI	Electrospray ionization
HGB	Hemoglobin count
GGO	Ground-glass opacity
Healthy→ILAs subjects	The subjects who were disease-free initially and then one year later suffered from ILAs
HRCT	High-resolution computed tomography
ILAs	Interstitial lung abnormalities
ILD	Interstitial lung disease
IPA	Ingenuity pathway analysis
IPF	Idiopathic pulmonary fibrosis
LC-Q-TOF-MS	Liquid chromatography quadruple time-of-flight mass spectrometry
LY	Lymphocyte count
mTOR	Mammalian target of rapamycin
NE	Neutrophil count
NSCLC	Non-small-cell lung cancer
PA	Phosphatidic acid
PC	Phosphatidylcholine
PCA	Principal component analysis
PE	Phosphatidylethanolamine
PI3K	Phosphatidyl inositol 3-kinase
PLA	Phospholipase A
PLD	Phospholipase D
PLS-DA	Partial least squares discriminant analysis
PLT	Blood platelet count
PPI	Protein-protein interaction
QC	Quality control
RBC	Red blood cell count
RhoA	Ras homolog family member A
ROC	Receiver operating characteristic
RSDs	Relative standard derivations
RV	Representative value
sPLA	Secretory phospholipase A
SUA	Serum uric acid
WBC	White blood cell count
