# Supplementary material for: Potential Metabolic Biomarkers to Identify Interstitial Lung Abnormalities
Source: Int J Mol Sci. 2016 Jul 16;17(7):1148. doi: 10.3390/ijms17071148 (PMC4964521; doi:10.3390/ijms17071148)
Supplement: Supplementary file 1 [file ijms-17-01148-s001.pdf]

Supplementary Materials: Potential Metabolic Biomarkers to Identify Interstitial Lung Abnormalities

Yong Tan, Dongmei Jia, Zhang Lin, Baosheng Guo, Bing He, Cheng Lu, Cheng Xiao, Zhongdi Liu, Ning Zhao, Zhaoxiang Bian, Ge Zhang, Weidong Zhang, Xinru Liu and Aiping Lu

Table S1. Identified pathways associated with common metabolites.

n	Canonical Pathways	−log(*p*-Value)	Molecules
1	Phospholipases	5.52E+00	1-Acylglycerophosphocholine, phosphatidic acid, phosphatidylcholine
2	Triacylglycerol Biosynthesis	5.26E+00	1-Acylglycerophosphocholine, phosphatidic acid, phosphatidylcholine
3	RhoA Signaling	4.95E+00	Phosphatidic acid, phosphatidylcholine
4	p70S6K Signaling	3.78E+00	Phosphatidic acid, phosphatidylcholine
5	mTOR Signaling	3.78E+00	Phosphatidic acid, phosphatidylcholine
6	Phospholipase C Signaling	3.51E+00	Phosphatidic acid, phosphatidylcholine
7	Gαq Signaling	3.51E+00	Phosphatidic acid, phosphatidylcholine
8	Choline Biosynthesis III	3.40E+00	Phosphatidic acid, phosphatidylcholine
9	Endothelin-1 Signaling	2.94E+00	Phosphatidic acid, phosphatidylcholine
10	Phosphatidylethanolamine Biosynthesis	1.83E+00	Phosphatidylethanolamine
11	Choline Degradation I	1.73E+00	Betaine aldehyde
